# Saliency-Driven Hand Gesture Recognition Incorporating Histogram of Oriented Gradients (HOG) and Deep Learning

**DOI:** 10.3390/s23187790

**Published:** 2023-09-11

**Authors:** Farzaneh Jafari, Anup Basu

**Affiliations:** Department of Computing Science, University of Alberta, Edmonton, AB T6G 2E8, Canada

**Keywords:** Canny edge detection, convolutional neural network (CNN), hand gesture detection, histogram of oriented gradients (HOG), saliency map, skin color

## Abstract

Hand gesture recognition is a vital means of communication to convey information between humans and machines. We propose a novel model for hand gesture recognition based on computer vision methods and compare results based on images with complex scenes. While extracting skin color information is an efficient method to determine hand regions, complicated image backgrounds adversely affect recognizing the exact area of the hand shape. Some valuable features like saliency maps, histogram of oriented gradients (HOG), Canny edge detection, and skin color help us maximize the accuracy of hand shape recognition. Considering these features, we proposed an efficient hand posture detection model that improves the test accuracy results to over 99% on the NUS Hand Posture Dataset II and more than 97% on the hand gesture dataset with different challenging backgrounds. In addition, we added noise to around 60% of our datasets. Replicating our experiment, we achieved more than 98% and nearly 97% accuracy on NUS and hand gesture datasets, respectively. Experiments illustrate that the saliency method with HOG has stable performance for a wide range of images with complex backgrounds having varied hand colors and sizes.

## 1. Introduction

Gestures are used for human interaction to express feelings, communicate non-verbal information, and increase the value of messages. A gesture can be an intuitive human–computer interface that helps machines understand body language for various purposes. Both online and offline applications, such as interacting with a computer, recognizing pedestrians and police hand signs in automated cars, gesture-based game control, and medical operations that use this technology are still in their infancy.

Two main approaches to detecting hand gestures are glove-based analysis and vision-based analysis. Glove-based techniques take advantage of sensors attached directly to the glove and accurately analyze hand movements. Vision-based methods can help users feel more comfortable without annoying physical limitations. They utilize a camera(s) to capture human hand signs and provide a more natural posture. The most essential ability of vision-based techniques is filtering out irrelevant and complex information and considering the most useful information during detection.

In this paper, we propose a general method of hand gesture recognition based on computer vision methods and compare the empirical results of input images with complex backgrounds. Recognizing different hand signs using an integrated structure based on saliency maps and histogram of oriented gradients (HOG) creates a filter for selecting target regions by ignoring irrelevant information. This leads to an increase in the performance of gesture recognition algorithms. These methods detect the exact regions of hand gestures and ignore complex backgrounds from input images. Lastly, we improve a convolutional neural network (CNN) with two blocks to identify hand postures for increasing accuracy and stability. We use the NUS Hand Posture Dataset II and the hand gesture dataset to demonstrate the performance of our model. In our experiments, we applied six diverse aggressive types of noise such as Gaussian, impulse, Laplacian, multiplicative-Gaussian, Poisson, and uniform to around 60% of our datasets to evaluate our model’s performance while encountering low-quality images.

The remainder of this paper is organized as follows. Section 2 presents some related work on hand gesture detection. Section 3 describes the framework of the proposed model for image saliency with the HOG model. Section 4 describes the performance evaluation. Section 5 provides a brief conclusion.

## 2. Related Work

Ajallooeian et al. [1] used a saliency-based model of visual attention to find potential hand regions in video frames. The saliency maps of the differences between consecutive video frames are overlaid to obtain the overall movement of the hand. A Loci feature extraction method is used to obtain hand movement. Then, the extracted feature vector is used for training an SVM to classify the postures. Chuang et al. [2] proposed a model that integrated image saliency and skin color information to improve the performance of the hand gesture detection model, with SVM utilized to classify hand gestures. Zhang et al. [3] built up a method based on saliency and skin color detection algorithms, including a pixel-level hand detection method, region-level hand detection method, and a multiple saliency map fusion framework that achieves the deep integration of the bottom-up saliency and top-down skin color information. This method has excellent performance and is reliable against complex backgrounds. A saliency detection method using a top-down dark channel prior is developed to determine the hand location and contour of the gesture. Then, it is integrated with a graph-based segmentation approach to make a final confidence map for segmentation [4].

Zamani and Rashidy [5] after extracting the saliency map used principal component analysis (PCA) and linear discriminant analysis (LDA) in order to reduce dimension, minimize class external similarity, and maximize class internal similarity, which led to the accuracy reaching 99.88% using a 4-fold cross-validation. Yin and Davis [6] developed a gesture salience method and a gesture spotting and recognition method based on hand tracking and concatenated hidden Markov models. Schauerte and Stiefelhagen [7] trained a conditional random field to combine relevant features to multi-scale spectral saliency, salient object detection, probabilistic pointing cone, and probabilistic target maps to highlight image regions highly similar to the target object. Reducing the false positive rate in skin segmentation using saliency detection is a method that was proposed by Santos et al. [8]. The weighted image is considered as input for the saliency detector, and the probability map is used to prevent discarding skin pixel adjustment to the boundary list. When it comes to using superpixel in the implementation of the saliency map, it can easily be replaced with a superpixel structure.

Vishwakarma et al. [9] detected hand gestures in static hand posture images by following these steps: (a) segmentation of hand region, (b) applying the saliency method, and (c) extracting Gabor and pyramid histogram of oriented gradients (PHOG). The Gabor filter extracts the texture features at different orientations, and PHOG extracts the shape of the hand by calculating the spatial distribution of the skin saliency image. Finally, extracted features are classified by a support vector machine (SVM). The method based on RGB-D data is proposed to deal with large-scale videos to achieve gesture shape recognition. The inputs are expanded into 32-frame videos to learn details better, and the RGB and depth videos are sent to the C3D model to extract spatiotemporal features, which combine together to boost the performance of the model and avoid unreasonable synthetic data to the uniform dimension of C3D features [10].

Yang et al. [11] proposed saliency-based features and sparse representations for hand posture recognition utilizing sparsity term parameters and sparse coefficient computation. The histogram intersection kernel function was employed to deal with non-linear feature maps by mapping the original features into the kernel feature space and using sparse representation classification in the kernel of the feature space. The fast saliency model with a 5×5 kernel convolution was proposed to obtain the saliency map of the input images. Candidate regions are extracted from the saliency map using adaptive thresholding, connected domain filtering, and the HOG descriptor for each area [12].

A two-stage hand gesture recognition is proposed to support a patient assistant system. The first step utilizes a saliency map to simplify hand gesture detection, and the second step classifies the patient’s postures. A novel combined loss function and a kernel-based channel attention layer are used to optimize the saliency detection model and emphasize salient features, respectively [13]. Guo et al. [14] proposed a motion saliency model based on a hierarchical attention network for action detection. They also defined combination schemes to link the attention and base branches to explore their impacts on the model. Regarding the characteristics of visual and thermal images, Xu et al. [15] integrate CNN feature and saliency map fusion methods to achieve RGB-T salient object recognition. In this method, the salient object is separated from the background with a fine boundary, and the noise inside a salient object is effectively suppressed.

Ma et al. [16] designed hand joint-based recognition based on a neural network and noisy datasets. To promote the availability of this model with noisy datasets, a nested interval unscented Kalman filter (UKF) with long-term and short-term memory (NIUKF-LSTM) network is proposed to improve the performance of the proposed model when dealing with noisy images. Evaluating the perceptual quality assessment owing to the quality degradation plays a vital role in visual communication systems. The quality assessment in such systems can be performed subjectively and objectively, and the objective quality assessment is taken into account thanks to its high efficiency and easy implementation [17]. Since computer-generated screen content has many characteristics different from camera-captured scene content, estimating the quality of experiment (QoE) in various screen content is a piece of essential information for improving communication systems [18]. The full-reference image quality assessment (IQA) metrics evaluate the distortion of an image generally by measuring its deviation from a reference or high-quality image. The reduced-reference and no-reference IQA metrics are used when the reference image is not fully available. In this case, some characteristics are driven by a perfect-quality image, and the distorted image’s deviation can be measured from these characteristics [19,20,21].

## 3. Proposed Method

**Figure 1:**
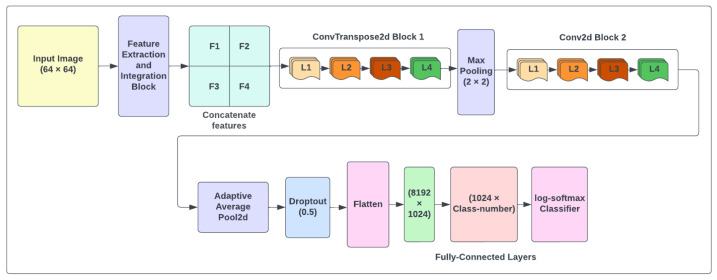
Architecture of the proposed method.

We introduce a method that eliminates the complexity of image backgrounds using features extracted from original images and binary operators. Detecting objects in complicated scenes is one of the challenging tasks in hand gesture recognition since it is difficult to recognize the intent object among many others. The proposed model provides an efficient system based on deep learning for recognizing the structure of hand postures in complex backgrounds by developing the architecture shown in Figure 1.

**Figure 2:**
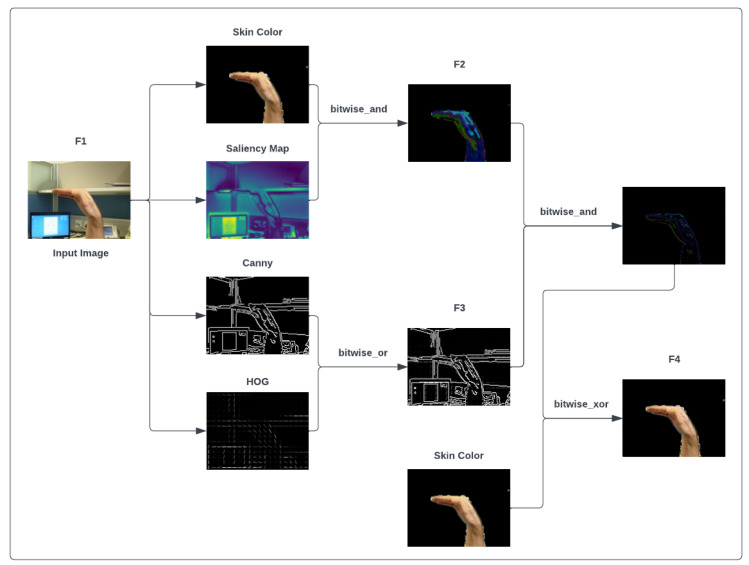
The proposed feature extraction and integration block. Extracting and integrating saliency map, skin color, HOG, and Canny features from the NUS Hand Posture Dataset II images using bitwise operators for static hand gesture recognition.

In this architecture, the size of the input image is equal to 64×64, which is given to the feature extraction and integration block as an input. Once the features have been extracted from the original images, the bitwise operators can mix these features to distinguish more details from hand-shaped textures. Figure 2 shows the process of the proposed feature extraction and integration model (see Appendix A). First, skin color [22], saliency [23], Canny [24], and HOG [25,26] features are extracted from the original image. The bitwise AND operator combines skin color and saliency feature maps, which gives us a new feature map. Using the bitwise OR operator, we perform a similar action for Canny edge detection and HOG features. Then, the two mixed feature maps produced by the previous steps are combined by the bitwise AND operator to make an exact region of hand shape, and the final result is mixed with skin color by the bitwise XOR operator to add hand region to the skin color information. Eventually, the output feature maps (F1, F2, F3, and F4) are given to the next block for concatenation. The F1–F4 features are represented by Equations (1)–(4):

(1) $$F1=O_{i}$$

(2) $$F2=F_{SC}\wedge F_{S}$$

(3) $$F3=F_{C}\vee F_{HOG}$$

(4) $$F4=\left(\left(F_{SC}\wedge F_{S}\right)\right)\wedge \left(F_{C}\vee F_{HOG}\right))\;⊕\;F_{SC}$$

where $O_{i}$ is an original image; $F_{SC}$, $F_{S}$, $F_{C}$, and $F_{HOG}$ are skin color, saliency, Canny, and histogram of oriented gradient features, respectively; and F1, F2, F3, and F4 represent output features of the feature extraction and integration block. In the next step, all extracted features are concatenated and used as input for the classification section.

Table 1 demonstrates the improved CNN model summary used for classification. The total number of trainable parameters in this architecture is 9,026,502. As indicated in this table, there are two convolutional blocks, each with four layers. We utilized ConvTranspose2d with batch normalization and rectified linear unit (ReLU) activation in the first block in each layer. The padding and stride value is one, and the kernel size is three. The ConvTranspose2d layers are considered as the gradient of Conv2d and are used for creating features. In the second block, we used Conv2d instead of ConvTranspose2d layer with the same parameters to shrink our output to detect features. After each block, 2D MaxPooling reduces computational complexity in order to detect features in the feature maps. The fully connected layer with Flog-softmax is used to classify hand shapes.

## 4. Experiments

In this section, experiments are designed to evaluate the performance of the saliency map incorporating the HOG features.

### 4.1. Datasets

**Figure 3:**
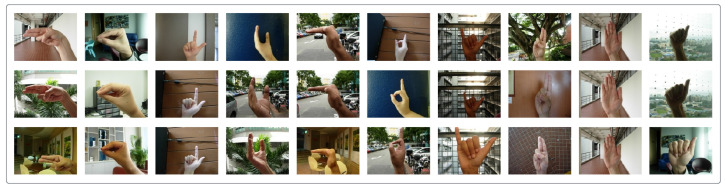
Sample images from the NUS dataset with complex backgrounds.

**Figure 4:**
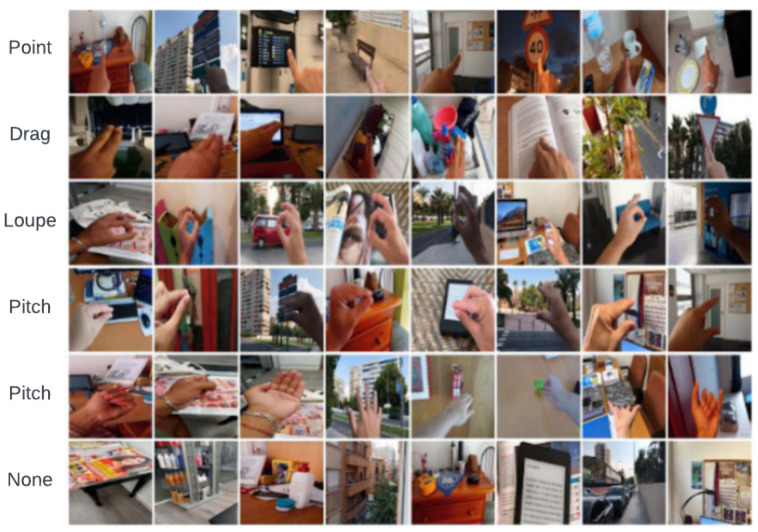
Sample images from the hand gesture dataset with complex backgrounds.

As can be seen in Figure 3 and Figure 4, two different types of datasets like the NUS Hand Posture Dataset II [27] and the hand gesture dataset (real samples) [28] have been used. The NUS and hand gesture datasets contain 2000 and 12,064 images of diverse hand gestures with different backgrounds, respectively. The NUS dataset contains A to J alphabets (10 classes) captured by different hand sizes and scenes. The hand gesture dataset contains six diverse groups: drag, loupe, none, other, point, and scale are captured under different and complex backgrounds, making the dataset more challenging.

### 4.2. Implementation Details

This study uses Python 3.7.12 with CUDA version 11.8.89 for all our experiments. The experiments have been carried out using PyTorch, an open-source and optimized tensor library for deep learning [29]. The model is trained at each stage with batch size 32, a learning rate of 0.0002, and a dropout of 0.5. We use a cross-entropy loss function, Stochastic Gradient Descent (SGD) optimizer, and train with NVIDIA GeForce RTX 2080 SUPER (NVIDIA, Santa Clara, CA, USA) [30]. Given the GPU limitation, we resize images to 64×64. In this experiment, we considered 80% of total data for training, 10% for validation, and 10% for testing, which is randomly selected from the whole dataset.

### 4.3. Analysis

**Figure 5:**
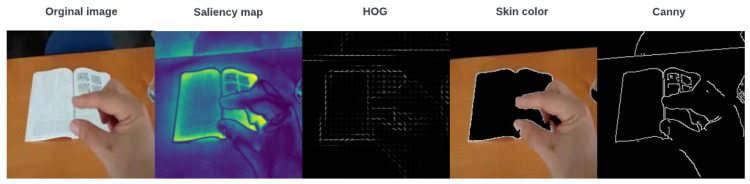
Different extracted features from the hand gesture dataset.

**Figure 6:**
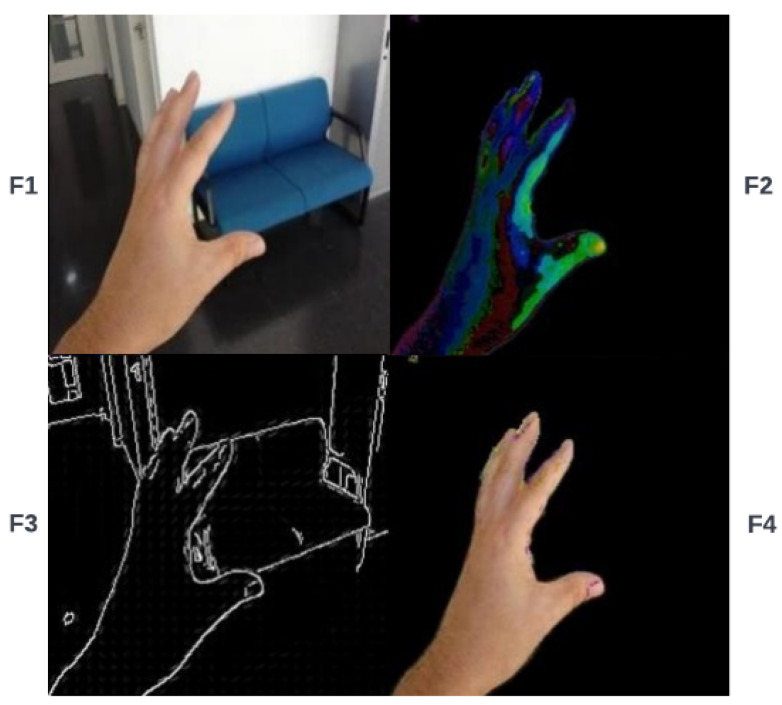
Proposed feature map of the hand gesture dataset. F1, F2, F3, and F4 are obtained from the extraction and integration block.

In the experiments, four main features, namely saliency map, skin color, histogram of oriented gradients (HOG), and Canny edge detection, are extracted from the main input image. Figure 5 shows different extracted features from the original image. The proposed features (F1, F2, F3, and F4) shown in Figure 6 are extracted from the main image in the extraction and integration block, and an improved CNN model recognizes different hand shapes with complex scenes. It can be seen from Table 2 that the performance of the obtained features with 99.78% in the NUS dataset and 97.21% in the hand gesture dataset is higher than other single features that have been used in classification.

**Figure 7:**
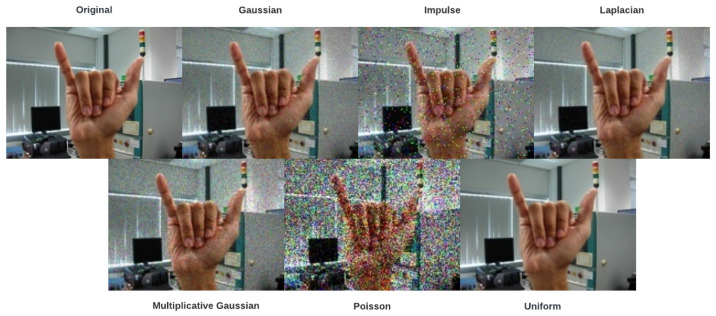
Original image and six others with various types of noise on the hand gesture dataset.

We apply some aggressive image noises randomly in 60% of our datasets to alleviate the problem of insufficient training and testing data. The six types of image noises applied to our datasets are namely Gaussian, impulse, Laplacian, multiplicative-Gaussian, Poisson, and uniform. The real rate of noise distribution in all functions is 0.9 except for impulse, which is equal to 0.1 [31] Figure 7 shows six image noises of the aforementioned applied on an original image.

Some of the referenced-based image quality estimation metrics such as mean square error (MSE), global relative error (ERGAS), multi-scale structural similarity index (MSSSIM), peak signal-to-noise ratio (PSNR), root mean squared error (RMSE), spectral angle mapper (SAM), structural similarity index (SSIM), universal quality image index (UQI), and visual information fidelity (VIF) are estimated for six different image noises mentioned above (Gaussian, impulse, Laplacian, multiplicative-Gaussian, Poisson, and uniform) which are applied on both the NUS Hand Posture Dataset II (Table 3) and the hand gesture dataset (Table 4). From a representation perspective, MSSSIM, SAM, SSIM, UQI, and VIFP are normalized, but MSE, EGRAS, PSNR, and RMSE are not. Therefore, the normalized IQA metrics can be treated as more understandable than other assessments. Applying these noises to our datasets can obviously show the performance of our proposed model against input noisy visual data. Table 5 shows that the accuracy of the NUS Hand Posture Dataset II with 98.83% and the hand gesture dataset with 96.63% is only reduced by around 1% than using datasets with perfect-quality images.

### 4.4. Discussion

We compared the proposed method with three state-of-the-art pyramid pooling and saliency detection methods, which include CNN-SPP [40] and saliency with skin color information [2]. The dataset used in all the above-mentioned methods is the NUS Hand Posture Dataset II, and it contains human hand gestures with different sizes and skin color information. The CNN-SPP [40] has two convolutional blocks with four layers in each. The spatial pyramid pooling (SPP) is extracted from the last layer of each block. The fully connected network contains two layers with 8192 neurons fully connected to 1024 neurons in the first layer and 1024 neurons fully connected to the number of classes of neurons in the next layer, and includes a softmax classifier.

Chuang [2] proposed another method to detect hand gestures in complex backgrounds. In this method, some features like a saliency map and skin color features are extracted from the image. These features help in identifying the hand gesture and adopt a visual cortex-based feature extraction method. Then, a linear SVM is used to recognize the hand posture according to the results of hand area detection, improving the result to around 95%. In this method, an isophote-based operator is used to capture the potential structure and global saliency information of each pixel. The potential structure is used to calculate the center-surround contrast and combined with the global saliency map to compute the final saliency map.

A hand-gesture-controlled PAS proposed in [13] uses a two-stage hand recognition architecture to integrate the convolution and transformer architectures. This method designed a saliency detection method to overcome some challenges that exist in vision-based approaches like occlusion, varying illumination, background diversity, and the detection of skin regions. The saliency map obtains the exact hand region of hand shape to be fed into the classification network. The AKCAL network in this architecture emphasizes the features relevant to classification. The recognition accuracy for the NUS dataset in this method is equal to 98.0%.

**Figure 8:**
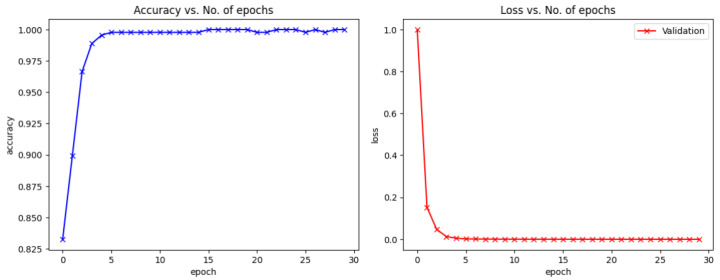
The performance of the proposed model for the NUS Dataset II.

The NUS hand posture images with varying backgrounds, hand sizes, and skin colors are very challenging hand postures to identify. As can be seen in Table 6, our proposed method performs much better than Tan’s [40] and Chuang’s [2] approaches to detecting hand postures with complex backgrounds in the NUS Hand Posture Dataset II. Figure 8 shows the validation loss and validation accuracy of the proposed model on the NUS dataset without noise. Based on the learning curves, it is obvious that the validation accuracy keeps increasing and validation loss keeps decreasing.

## 5. Conclusions and Future Work

We introduced a novel method integrating the histogram of oriented gradients (HOG), skin color, Canny edge detection, and saliency maps using bitwise operators to detect hand postures with complex scenes by an improved CNN model. Using integrated feature maps identified the exact regions of the gestures in each input image and increased the accuracy. Apart from this, the proposed method enabled distinguishing postures better given complex backgrounds. The NUS hand posture II and the hand gesture datasets were used in the experiment, and the results showed that the proposed method improved the performance of hand gesture recognition in these datasets with and without image noises.

In our future work, we will address issues with the quality evaluation of image dehazing methods in vision-based hand gesture recognition systems. The image quality of experiments (QoE) is an essential aspect of various intelligent systems like those detecting hand postures since low-quality images or videos can have an adverse effect on identification performance. Evaluating gesture detection by incorporating audio-visual saliency will be considered in the next step of recognizing hand gestures.

## Figures and Tables

**Table 1:** Summary of the improved CNN architecture with an input size of 64 × 64.

Stage	Layer (Type:Depth-Index)	Output Size	Param
Input	-	[−1, 3, 32, 32]	-
Block 1	ConvTranspose2d:1–1	[−1, 64, 32, 32]	1792
	BatchNorm2d:1–2	[−1, 64, 32, 32]	128
	ReLU:1–3	[−1, 64, 32, 32]	-
	ConvTranspose2d:1–4	[−1, 64, 32, 32]	36,928
	BatchNorm2d:1–5	[−1, 64, 32, 32]	128
	ReLU:1–6	[−1, 64, 32, 32]	-
	ConvTranspose2d:1–7	[−1, 64, 32, 32]	36,928
	BatchNorm2d:1–8	[−1, 64, 32, 32]	128
	ReLU:1–9	[−1, 64, 32, 32]	-
	ConvTranspose2d:1–10	[−1, 64, 32, 32]	36,928
	BatchNorm2d:1–11	[−1, 64, 32, 32]	128
	ReLU:1–12	[−1, 64, 32, 32]	-
	MaxPool2d(2, 2):1–13	[−1, 64, 16, 16]	-
Block 2	Conv2d:1–14	[−1, 128, 16, 16]	73,856
	BatchNorm2d:1–15	[−1, 128, 16, 16]	256
	ReLU:1–16	[−1, 128, 16, 16]	-
	Conv2d:1–17	[−1, 128, 16, 16]	147,584
	BatchNorm2d:1–18	[−1, 128, 16, 16]	256
	ReLU:1–19	[−1, 128, 16, 16]	-
	Conv2d:1–20	[−1, 128, 16, 16]	147,584
	BatchNorm2d:1–21	[−1, 128, 16, 16]	256
	ReLU:1–22	[−1, 128, 16, 16]	-
	Conv2d:1–23	[−1, 128, 16, 16]	147,584
	BatchNorm2d:1–24	[−1, 128, 16, 16]	256
	ReLU:1–25	[−1, 128, 16, 16]	-
	AdaptiveAvgPool2d:1–26	[−1, 128, 8, 8]	-
Fully connected layer	flatten	[−1, 8192]	-
	Linear(8192, 1024):1–27	[−1, 1024]	8,389,632
	ReLU:1–28	[−1, 1024]	-
	Linear(1024, class_number):1–29	[−1, 10]	6150
	F.log_softmax	[−1, 10]	-
Total params:	9,026,502		
Trainable params:	9,026,502		
Non-trainable params:	0		
Total mult-adds (M):	255.53		
Input size (MB):	0.01		
Forward/backward			
pass size (MB):	6.01		
Params size (MB):	34.43		
Estimated Total Size (MB):	40.45		

**Table 2:** Test accuracy results of the proposed model using the improved CNN model and different input features on NUS Hand Posture Dataset II and hand gesture dataset after 30 epochs.

Features	NUS Hand Posture Dataset II	Hand Gesture Dataset
Original images	97.27	94.50
Canny	94.92	92.96
Saliency	95.31	90.14
Skin color	96.88	95.42
HOG	97.66	92.07
Our proposed features	**99.78**	**97.21**

The bold shows the highest accuracy in each dataset.

**Table 3:** Reference-based image quality metrics to quantify the NUS Hand Posture Dataset II image quality after applying six diverse noises.

IQA Metrics	Gaussian	Impulse	Laplacian	Multiplicative-Gaussian	Poisson	Uniform
MSE [32]	62.22945	487.12366	37.50542	88.54241	1494.11719	10.68307
EGRAS [33]	53.44101	150.39209	41.77666	61.73005	242.83121	22.80766
MSSSIM [34]	0.97518	0.87680	0.98504	0.97033	0.77359	0.99655
PSNR [35]	30.19085	21.25441	32.38986	28.65929	16.38696	37.84384
RMSE [32]	7.88856	22.07088	6.12417	9.4097	38.65381	3.2685
SAM [36]	0.10161	0.26996	0.079261	0.11953	0.44247	0.042524
SSIM [37]	0.86747	0.56597	0.91653	0.84555	0.33831	0.98446
UQI [38]	0.97966	0.90523	0.98762	0.98508	0.83638	0.99668
VIFP [39]	0.45636	0.21459	0.53019	0.41593	0.11206	0.74939

**Table 4:** Reference-based image quality metrics to quantify the hand gesture dataset image quality after applying six diverse noises.

IQA Metrics	Gaussian	Impulse	Laplacian	Multiplicative-Gaussian	Poisson	Uniform
MSE [32]	58.9221	103.29271	31.5976	171.96166	1241.64085	3.43042
EGRAS [33]	23.54171	31.22135	17.26357	40.09161	107.35299	5.73945
MSSSIM [34]	0.9227	0.89105	0.9567	0.83608	0.54435	0.99596
PSNR [35]	30.42802	27.99011	33.13426	25.77649	17.19084	42.77733
RMSE [32]	7.67607	10.1633	5.62117	13.11341	35.23692	1.85214
SAM [36]	0.053382	0.07099	0.038891	0.09115	0.24572	0.01163
SSIM [37]	0.61551	0.55536	0.74782	0.424	0.12197	0.98187
UQI [38]	0.99473	0.99087	0.99711	0.9927	0.9396	0.99954
VIFP [39]	0.34229	0.31202	0.41475	0.28056	0.10697	0.73754

**Table 5:** Test accuracy results of the proposed model using the improved CNN model and different input features on NUS Hand Posture Dataset II and hand gesture dataset with 60% noise after 30 epochs.

Features	NUS Hand Posture Dataset II	Hand Gesture Dataset
Original images	94.53	94.76
Canny	80.47	88.00
Saliency	92.97	91.07
Skin color	94.14	93.82
HOG	94.14	89.27
Our proposed features	**98.83**	**96.63**

The bold shows the highest accuracy in each dataset.

**Table 6:** Comparison of state-of-the-art methods with our proposed method using the NUS Hand Posture Dataset II.

Models	Test Accuracy (%)
CNN-SPP model [40]	95.95
Saliency with skin color information [2]	95.27
Saliency with combined loss function [13]	98.00
Saliency with skin color information and HOG *	**99.78**

* Our proposed method. The bold shows the highest accuracy.

## Data Availability

Publicly available datasets were analyzed in this study. The datasets can be found here: NUS dataset: https://www.ece.nus.edu.sg/stfpage/elepv/NUS-HandSet (accessed on 1 November 2022); the hand gesture dataset (real samples): https://www.dlsi.ua.es/~jgallego/datasets/gestures/ (accessed on 15 May 2023).

## Appendix A

### Appendix A.1. Opencv Static Saliency Detection

OpenCV is an open-source library [41] used in computer vision applications. OpenCV provides a useful tool for extracting static saliency maps from input images. The saliency feature map is extracted from the standout part of an image so that neural networks and machine learning methods can easily focus on these points. One approach to detecting the saliency map is static saliency detection, in which algorithms detect salient objects in a static image using image features and statistics. Static saliency detection helps localize more interesting regions in an image. Some algorithms have already been implemented in OpenCV, but we only use the fine-grained method in our research.

The fine-grained algorithm is similar to human eyes with ganglion cells. On-center and off-center are two different types of ganglion cells. On-center focuses on more bright areas that are surrounded by a dark background. Off-center works in the opposite way: it concentrates on dark areas surrounded by a brighter background. The fine-grained algorithm computes the saliency map by considering on-center and off-center [23].

### Appendix A.2. Histogram of Oriented Gradients (HOG)

HOG is used in machine vision to detect objects by counting the occurrences of gradient orientation related to local picture parts. HOG concentrates on an object’s structure or shape. It uses the magnitude and considers the angle of the gradient to calculate the features for better performance. The image gradient is calculated by combining image magnitude and angle. First, Gx and Gy are calculated for each pixel using Equations (A1) and (A2):

(A1) $$G_{x}\left(r,c\right)=I\left(r,c+1\right)-I\left(r,c-1\right),$$

(A2) $$G_{y}\left(r,c\right)=I\left(r-1,c\right)-I\left(r+1,c\right),$$

where *r* and *c* refer to rows and columns of an input image. The magnitude and angle are calculated by Equations (A3) and (A4):

(A3) $$Magnitude\left(\mu \right)=\sqrt{G_{x}^{2}+G_{y}^{2}},$$

(A4) $$Angle\left(\theta \right)=|tan^{-1}\left(\frac{G_{y}}{G_{x}}\right)|,$$

The magnitude and angle matrices are segmented into 8 × 8 metrics. A nine-point histogram and nine-point bin (each bin has an angle range of 20 degrees) are calculated for each segment. As a block/segment includes 64 different values of magnitude and gradient, Equations (A5) and (A6) are used:

(A5) $$Number\;of\;bins=9(ranging\;from\;0^{\circ }\;\mathrm{to}\;180^{\circ }),$$

(A6) $$Stepsize\left(\Delta \theta \right)=\frac{180^{\circ }}{Number\;of\;bins=20^{\circ }},$$

The boundaries of the $J_{th}$ bin is obtained by Equation (A7):

(A7) [Δθ.j,Δθ.(j+1)],

The center of each bin is calculated by Equation (A8):

(A8) $$C_{j}=\Delta \theta \left(j+0,5\right),$$

For calculating the $J_{th}$ bin and ${\left(J+1\right)}_{th}$ bin, we use Equations (A9)–(A11):

(A9) $$j=\lfloor \left(\frac{\theta }{\Delta \theta }-\frac{1}{2}\right)\rfloor ,$$

(A10) $$V_{j}=\mu .\left[(\frac{\theta }{\Delta \theta }-\frac{1}{2})\right],$$

(A11) $$V_{j+1}=\mu .\left[(\frac{\theta -C_{j}}{\Delta \theta )}\right],$$

For each block, an array is considered to be a bin. Then, $V_{j}$ and $V_{j+1}$ values are appended to it. After calculating the histogram for all blocks, four blocks from the nine-point histogram matrix are mixed with each other to create a new 2 × 2 block, resulting in a vector with 36 features (Equation (A12)).

(A12) $$f_{bi}=\left[b_{1},b_{2},b_{3},...,b_{36}\right],$$

The L2 norm is used to normalize all the values in the $f_{b}$ vector (Equation (A13)):

(A13) $$f_{bi}=\frac{f_{bi}}{\sqrt{\left|\right|f_{bi}{\left|\right|}^{2}+\varepsilon }}.$$

This normalization leads to reducing the contrast changes between images of the same object. This vector is calculated for each block and HOG features for each image are obtained [25,26].

### Appendix A.3. Canny Edge Detection

The Canny edge detector can find the edges of images, similar to the way that human eyes can analyze image details and determine them in milliseconds. This algorithm can detect the edges following the steps of noise reduction, gradient calculation, non-maximum suppression, double threshold, and edge tracking by hysteresis. First, a grayscale filter is applied to the original image, and a Gaussian blur is used to reduce background noise by smoothing it. The image convolution technique is applied with a Gaussian kernel. A Gaussian filter kernel of (2k+1)(2k+1) is obtained by Equation (A14):

(A14) $$H_{ij}=\frac{1}{2\pi \sigma^{2}}exp\left(-\frac{(i-{\left(k+1\right)}^{2}+{\left(j-\left(k+1\right)\right)}^{2}}{2\sigma^{2}}\right),$$

1≤i,j≤(2k+1),

The edge intensity and direction are calculated by the image gradient. Edges are affected by a change in pixel intensity. By applying filters the intensity is highlighted in both horizontal and vertical directions. Then, the derivatives of *x* and *y* are calculated by Sobel kernels Kx and Ky. The magnitude and angle of the gradient are calculated by Equations (4) and (A1). The goal of the non-maximal suppression algorithm is to create thinner edges. It works when we consider all points of the gradient intensity matrix and try to identify pixels with maximum values in the edge directions. The double threshold step tries to detect three kinds of pixels: strong, weak, and irrelevant. The pixels with high intensity are classified as strong pixels. The weak pixels are those with an intensity that is not as high as that of strong pixels, but weak pixels are also not too small. Other pixels are considered irrelevant for the edge. Strong pixels and weak pixels are considered high thresholds and low thresholds, respectively. The weak pixels are all pixels between the low and high thresholds. The hysteresis mechanism helps us find irrelevant and strong pixels and transform weak pixels into strong pixels if and only if one of the neighbor pixels is strong [24].

### Appendix A.4. Color Space for Skin Color Detection

The color space method for skin color is a mathematical model that considers the information from about three or four colors for skin detection. There are different models to detect skin color. Transforming RGB to a normalized RGB color space is achieved by the normalization process (Equations (A15)–(A18)):

(A15) $$r=\frac{R}{R+G+B},$$

(A16) $$g=\frac{G}{R+G+B},$$

(A17) $$b=\frac{B}{R+G+B},$$

(A18) r+g+b=1.

The HSV (which stands for hue, saturation, value) color space is an alternative version of the RGB model. The conversion from RGB to HSV takes time and is expensive. If there are large fluctuations in the color information, like hue and saturation, pixels that have small and large intensities are not considered. Transforming color images in the RGB color space to the HSV color space is achieved by the formulae (Equations (A19)–(A21)):

(A19) $$H=arccos(\frac{\frac{1}{2}\left(2R-G-B\right)}{\sqrt{{\left(R-G\right)}^{2}-\left(R-B\right)\left(G-B\right)}}),$$

(A20) $$S=\frac{\left(max(R,B,G)-min(R,B,G)\right)}{max(R,B,G)},$$

(A21) V=max(R,G,B).

This method is convenient for detecting human faces and hands in color images [22].

## References

[1] Ajallooeian M., Borji A., Araabi B.N., Ahmadabadi M.N., Moradi H. (2009). Fast hand gesture recognition based on saliency maps: An application to interactive robotic marionette playing. Proceedings of the RO-MAN 2009-The 18th IEEE International Symposium on Robot and Human Interactive Communication.

[2] Chuang Y., Chen L., Chen G. (2014). Saliency-guided improvement for hand posture detection and recognition. Neurocomputing.

[3] Zhang Q., Yang M., Kpalma K., Zheng Q., Zhang X. (2018). Segmentation of hand posture against complex backgrounds based on saliency and skin colour detection. IAENG Int. J. Comput. Sci..

[4] Zhang Q., Yang M., Zheng Q., Zhang X. (2017). Segmentation of hand gesture based on dark channel prior in projector-camera system. Proceedings of the 2017 IEEE/CIC International Conference on Communications in China (ICCC).

[5] Zamani M., Kanan H.R. (2014). Saliency based alphabet and numbers of American sign language recognition using linear feature extraction. Proceedings of the 2014 4th International Conference on Computer and Knowledge Engineering (ICCKE).

[6] Yin Y., Davis R. Gesture spotting and recognition using salience detection and concatenated hidden markov models. Proceedings of the 15th ACM on International Conference on Multimodal Interaction.

[7] Schauerte B., Stiefelhagen R. (2014). “Look at this!” learning to guide visual saliency in human-robot interaction. Proceedings of the 2014 IEEE/RSJ International Conference on Intelligent Robots and Systems.

[8] Santos A., Pedrini H. (2015). Human skin segmentation improved by saliency detection. Proceedings of the Computer Analysis of Images and Patterns: 16th International Conference, CAIP 2015.

[9] Vishwakarma D.K., Singh K. (2016). A framework for recognition of hand gesture in static postures. Proceedings of the 2016 International Conference on Computing, Communication and Automation (ICCCA).

[10] Li Y., Miao Q., Tian K., Fan Y., Xu X., Li R., Song J. (2016). Large-scale gesture recognition with a fusion of rgb-d data based on the c3d model. Proceedings of the 2016 23rd International Conference on Pattern Recognition (ICPR).

[11] Yang W., Kong L., Wang M. (2016). Hand gesture recognition using saliency and histogram intersection kernel based sparse representation. Multimed. Tools Appl..

[12] Qi S., Zhang W., Xu G. Detecting consumer drones from static infrared images by fast-saliency and HOG descriptor. Proceedings of the 4th International Conference on Communication and Information Processing.

[13] MacDorman K.F., Laskar R.H. (2023). Patient Assistance System Based on Hand Gesture Recognition. IEEE Trans. Instrum. Meas..

[14] Guo Z., Hou Y., Xiao R., Li C., Li W. (2023). Motion saliency based hierarchical attention network for action recognition. Multimed. Tools Appl..

[15] Xu C., Li Q., Zhou M., Zhou Q., Zhou Y., Ma Y. (2022). RGB-T salient object detection via CNN feature and result saliency map fusion. Appl. Intell..

[16] Ma C., Wang A., Chen G., Xu C. (2018). Hand joints-based gesture recognition for noisy dataset using nested interval unscented Kalman filter with LSTM network. Vis. Comput..

[17] Zhai G., Min X. (2020). Perceptual image quality assessment: A survey. Sci. China Inf. Sci..

[18] Min X., Gu K., Zhai G., Yang X., Zhang W., Le Callet P., Chen C.W. (2021). Screen content quality assessment: Overview, benchmark, and beyond. ACM Comput. Surv..

[19] Min X., Gu K., Zhai G., Liu J., Yang X., Chen C.W. (2017). Blind quality assessment based on pseudo-reference image. IEEE Trans. Multimed..

[20] Min X., Zhai G., Gu K., Liu Y., Yang X. (2018). Blind image quality estimation via distortion aggravation. IEEE Trans. Broadcast..

[21] Min X., Ma K., Gu K., Zhai G., Wang Z., Lin W. (2017). Unified blind quality assessment of compressed natural, graphic, and screen content images. IEEE Trans. Image Process..

[22] Shaik K.B., Ganesan P., Kalist V., Sathish B.S., Jenitha J.M.M. (2015). Comparative study of skin color detection and segmentation in HSV and YCbCr color space. Procedia Comput. Sci..

[23] Saliency API, OpenCV. https://docs.opencv.org/4.x/d8/d65/group-saliency.html.

[24] Sahir S. (2019). Canny Edge Detection Step by Step in Python—Computer Vision. https://towardsdatascience.com/Canny-edge-detection-step-by-step-in-python-computer-vision-b49c3a2d8123.

[25] Dalal N., Triggs B. (2005). Histograms of oriented gradients for human detection. Proceedings of the 2005 IEEE Computer Society Conference on Computer Vision and Pattern Recognition (CVPR’05).

[26] Tyagi M. (2021). HOG(Histogram of Oriented Gradients). https://towardsdatascience.com/hog-histogram-of-oriented-gradients-67ecd887675f.

[27] The NUS Hand Posture Dataset-II. (n.d.). https://www.ece.nus.edu.sg/stfpage/elepv/NUS-HandSet/.

[28] Hand Gestures Dataset. https://www.dlsi.ua.es/~jgallego/datasets/gestures/.

[29] Paszke A., Gross S., Massa F., Lerer A., Bradbury J., Chanan G., Killeen T., Lin Z., Gimelshein N., Antiga L. (2019). PyTorch: An Imperative Style, High-Performance Deep Learning Library. Advances in Neural Information Processing Systems 32.

[30] NVIDIA GeForce RTX 2080 SUPER. https://www.nvidia.com/en-us/geforce/news/gfecnt/nvidia-geforce-rtx-2080-super-out-now/.

[31] Wand.Image—Image Objects. https://docs.wand-py.org/en/0.6.2/wand/image.html.

[32] Søgaard J., Krasula L., Shahid M., Temel D., Brunnström K., Razaak M. Applicability of existing objective metrics of perceptual quality for adaptive video streaming. Proceedings of the Electronic Imaging, Image Quality and System Performance XIII.

[33] Renza D., Martinez E., Arquero A. (2012). A new approach to change detection in multispectral images by means of ERGAS index. IEEE Geosci. Remote Sens. Lett..

[34] Nasr M.A.-S., AlRahmawy M.F., Tolba A.S. (2017). Multi-scale structural similarity index for motion detection. J. King Saud-Univ.-Comput. Inf. Sci..

[35] Deshpande R.G., Ragha L.L., Sharma S.K. (2018). Video quality assessment through PSNR estimation for different compression standards. Indones. J. Electr. Eng. Comput. Sci..

[36] Li X., Jiang T., Fan H., Liu S. (2023). SAM-IQA: Can Segment Anything Boost Image Quality Assessment?. arXiv.

[37] Wang Z., Simoncelli E.P., Bovik A.C. (2003). Multiscale structural similarity for image quality assessment. Proceedings of the Thrity-Seventh Asilomar Conference on Signals, Systems & Computers.

[38] Egiazarian K., Astola J., Ponomarenko N., Lukin V., Battisti F., Carli M. New full-reference quality metrics based on HVS. Proceedings of the Second International Workshop on Video Processing and Quality Metrics.

[39] Wu J., Lin W., Shi G., Liu A. (2013). Reduced-reference image quality assessment with visual information fidelity. IEEE Trans. Multimed..

[40] Tan Y.S., Lim K.M., Tee C., Lee C.P., Low C.Y. (2021). Convolutional neural network with spatial pyramid pooling for hand gesture recognition. Neural Comput. Appl..

[41] Bradski G. (2000). The openCV library. Dobb’s J. Softw. Tools Prof. Program..

